# Case Report: Hypothalamic Amenorrhea Following COVID-19 Infection and Review of Literatures

**DOI:** 10.3389/fendo.2022.840749

**Published:** 2022-06-10

**Authors:** Paolo Facondo, Virginia Maltese, Andrea Delbarba, Ilenia Pirola, Mario Rotondi, Alberto Ferlin, Carlo Cappelli

**Affiliations:** ^1^ Department of Clinical and Experimental Sciences, Endocrine and Metabolic Unit, University of Brescia, Azienda Socio Sanitaria Territoriale (ASST) Spedali Civili Brescia, Brescia, Italy; ^2^ Unit of Internal Medicine and Endocrinology, Istituto Clinico Scientifico (ICS) Maugeri Istituto di Ricovero e Cura a Carattere Scientifico (I.R.C.C.S.), Laboratory for Endocrine Disruptors, University of Pavia, Pavia, Italy; ^3^ Unit of Andrology and Reproductive Medicine, Department of Medicine, University of Padova, Padova, Italy

**Keywords:** hypothalamic amenorrhea, COVID-19, hypothalamic–pituitary dysfunction, female hypogonadotropic hypogonadism, central amenorrhea

## Abstract

SARS-CoV-2 infection, responsible for the coronavirus disease 2019 (COVID-19), can impair any organ system including endocrine glands. However, hypothalamic–pituitary dysfunctions following SARS-CoV-2 infection remain largely unexplored. We described a case of hypothalamic amenorrhea following SARS-CoV-2 infection in a 36-year-old healthy woman. The diagnostic workup excluded all the causes of secondary amenorrhea, in agreement to the current guidelines, whereas the gonadotropin increase in response to GnRH analogue tests was suggestive for hypothalamic impairment. Therefore, since our patient did not present any organic cause of hypothalamic–pituitary disorder, we hypothesized that her hypothalamic deficiency may have been a consequence of SARS-CoV-2 infection. This assumption, besides on the temporal consecutio, is strengthened by the fact that SARS-CoV-2 infection can impair the hypothalamic circuits, altering the endocrine axes, given that angiotensin-converting enzyme 2 receptors have also been observed in the hypothalamus. We reviewed the literature regarding hypothalamic–pituitary dysfunction in patients with SARS-CoV-2 infection. No study has previously described female hypogonadotropic hypogonadism with secondary amenorrhea following COVID-19. We suggest clinicians focusing greater attention on this possible endocrine disorder.

## Introduction

Coronavirus disease (COVID-19), caused by severe acute respiratory syndrome coronavirus 2 (SARS-CoV-2), is a primarily respiratory system disease, but it can lead to systemic manifestations, including in the cardiovascular, neurological, and gastrointestinal systems (1). Moreover, COVID-19 can also affect the endocrine system, since the angiotensin-converting enzyme 2 (ACE2) receptor—which is responsible together with transmembrane serine protease 2 (TMPRSS2) for the entry of SARS-CoV-2 to the cells—is also expressed in endocrine glands (thyroid, testis, ovary, adrenal, and pituitary) (2, 3). In addition, this virus can impair the hypothalamic circuits, altering the endocrine axes, since ACE2 receptors have also been observed in the hypothalamus (4). However, the endocrine complications, especially hypothalamic–pituitary dysfunction of COVID-19, are poorly reported and remain largely unexplored (5).

### Hypothalamic–Pituitary Dysfunction Following COVID-19: Review of Literatures

We present a case of hypothalamic amenorrhea following SARS-CoV-2 infection, and we reviewed the literature regarding hypothalamic–pituitary dysfunction of COVID-19 to evaluate if this possible dysfunction has already been reported.

Hypothalamic–pituitary dysfunctions following SARS-CoV-2 infection in adults have been described (**Table 1**) (6–29).

**Table 1:** Cases in literatures of hypothalamic–pituitary dysfunction after COVID-19 infection in adults.

Type of hypothalamic–pituitary dysfunction (with number of cases reported)	Case patient (M/F, yr, condition/comorbidities)	Time to onset of dysfunction after COVID-19 infection	Clinical presentation of hypothalamic–pituitary dysfunction	Outcome	Reference
Pituitary apoplexy (12 cases)	F, 28 yr, third trimester pregnant	Contextual to the infection	Headache, visual alterations, at pituitary imaging cystic-solid lesion with expanded sella and hemorrhage (suspected within a pregnancy enlargement of pituitary, doubtful preexisting adenoma)	TNS surgery after partum, with consequence of central hypothyroidism and central hypogonadism	Chan et al. (6)
	F, 44 yr, healthy	6 days	Headache, visual alterations, at pituitary imaging cystic-solid lesion with expanded sella and hemorrhage (suspected within a preexisting adenoma)	Refused surgery, central hypothyroidism	Ghosh et al. (7)
	F, 65 yr, healthy	Acute presentation		Hydrocortisone treatment, central hypothyroidism	Bordes et al. (8)
	M, 47 yr, healthy	2–3 weeks		TNS surgery, without complications	Santos et al. (9)
	M, 35 yr, healthy	Few days	Headache, at pituitary imaging recognition of hemorrhagic pituitary microadenoma with signs of pituitary apoplexy	Hospital monitoring, endocrinologic follow-up	LaRoy et al. (10)
	M, 46 yr, healthy	Acute presentation	Headache, at pituitary imaging recognition of hemorrhagic lesion and neuro-ophthalmic involvement	Corticosteroid therapy and discharge	Katti et al. (11)
	M, 27 yr, pre-existing Non-secreting pituitary macroadenoma	Acute presentation	Headache, visual alterations, at pituitary imaging cystic-solid lesion with expanded sella and hemorrhage in the context of a preexisting macroadenoma	Death for pulmonary complications	Solorio-Pineda et al. (12)
	M, 55 yr, T2DM, hypertension and pituitary macroadenoma resection 11 years ago, hormonal replacement therapy for panhypopituitarism	Acute presentation	Progressive decrease in visual acuity and oculomotor nerve palsy, at pituitary imaging enlarging residual pituitary adenoma with signs of pituitary apoplexy	TNS surgery, death for pulmonary complications	Kamel et al. (13)
	M, 75 yr, gastrointestinal disease	1 month	Headache, at pituitary imaging pituitary macroadenoma (previously undiagnosed) with signs of pituitary apoplexy	Hydrocortisone and thyroid replacement treatment, discharged in endocrinologic follow-up, follow-up pituitary imaging showed reduction in the size of the hemorrhagic lesion	Liew et al. (14)
	3 cases F, 54 yr, healthy; M, 56 yr, obese, hypertension, hypothyroidism; M, 56 yr, obese, hypertension	Few days	Headache, visual alterations and/or nerve palsies, at pituitary imaging cystic-solid lesion with expanded sella and hemorrhage (suspected within a preexisting adenoma, confirmed after surgery)	TNS surgery, with complete symptoms resolution and without complications; substitutive treatment with hydrocortisone and levothyroxine, and in the first patient also desmopressin; in the third patient consequence of central hypogonadism non yet treated	Martinez-Perez et al. (15)
SIADH (10 cases)	F, 70 yr, hypertension	Contextual to the infection	Signs of moderate-severe hyponatremia	Treatment of hyponatremia with clinical resolution	Uddin Chowdhury et al. (16)
	3 cases M, 58 yr, hypertension, asthma and dyslipidemia; M, 20 yr, healthy; M, 47 yr, healthy	Contextual to the infection	Signs of moderate-severe hyponatremia	Treatment of hyponatremia with clinical resolution	Yousaf et al. (17)
	2 cases F, 80 yr, healthy; M, 62 yr, healthy	Contextual to the infection	Signs of moderate-severe hyponatremia	Treatment of hyponatremia with clinical resolution	Ravioli et al. (18)
	M, 37 yr, healthy	Contextual to the infection	Signs of moderate-severe hyponatremia	Treatment of hyponatremia with clinical resolution	Sheikh et al. (19)
	M, 66 yr, T2DM, hypertension, hypothyroidism, and coronary insufficiency	Contextual to the infection	Signs of moderate-severe hyponatremia	Treatment of hyponatremia with symptoms resolution; hyponatremia persisted throughout follow-up and resolved spontaneously after 2 months	Saad et al. (20)
	M, 59 yr, healthy	Contextual to the infection	Hyponatremia with presyncope and sinus bradycardia	Treatment of hyponatremia with clinical resolution	Amir et al. (21)
	M, 75 yr, healthy	Likely contextual to the infection	Signs of hyponatremia with tonic–clonic seizures	Treatment of hyponatremia with clinical resolution	Ho et al. (22)
Central diabetes insipidus (with also central adrenal insufficiency in one case) (3 cases)	F, 60 yr, healthy	Eight weeks after COVID-19 infection	Polyuria, nocturia, polydipsia, craving for cold water, and hypernatremia; serum and urine osmolarity suggestive for diabetes insipidus; at brain imaging signs of infundibuloneuro hypophysitis	Oral desmopressin 0.1 µg twice a day, with resolution of symptoms	Misgar et al. (23)
	M, 28 yr, healthy	14 days	Myocarditis; polyuria, polydipsia, hypernatremia, and low urine osmolarity	Administration of 2 µg of desmopressin with clinical improvement	Sheikh et al (24),
	F, 44 yr, T2DM	12 days	Biochemical and dynamic test suggestive for central adrenal insufficiency (with dizziness, nausea, hypotension) and central diabetes insipidus (polyuria, polydipsia); normal pituitary and brain imaging	Hydrocortisone and desmopressin treatment, with clinical resolution	Sheikh et al. (25)
Central hypocortisolism (2 cases, with also Sheikh et al. (25))	M, 47 yr, T2DM	1 week	Biochemical exams suggestive for central hypocortisolism (with dyspepsia and eosinophilia)	Hydrocortisone treatment with clinical resolution, no spontaneous resolution of hypocortisolism 3 weeks after infection	Chua et al. (26)
Hypothalamic involvement with ophthalmoparesis or acute ischemic stroke or encephalitis (4 cases)	2 cases F, 60 yr, healthy; F, 35 yr, history of bulimia	10–15 days	Diplopia, headache, nerve palsy, or paresthesia and disorientation, at brain imaging hypothalamic alterations at cranial nerve nuclei	One month after infection resolution, diplopia and episodic memory loss persisted in the two patients, respectively.	Pascual-Goñi et al. (27)
	M, 73 yr, healthy	8 days	Acute ischemic stroke with hemiparesis and hypothalamic alterations at brain imaging	Consequences of stroke	Beyrouti et al. (28)
	F, 25 yr, healthy	Contextual to the infection	Anosmia, brain imaging suggestive for encephalitis with hypothalamic involvement	One month after infection, resolution of brain imaging and anosmia	Politi et al. (29)

M, male; F, female; yr, years; TNS, transnasosphenoidal; SIADH, syndrome of inappropriate antidiuretic hormone secretion; T2DM, type 2 diabetes mellitus.

The most frequently reported hypothalamic–pituitary alterations following COVID-19 are pituitary apoplexy and the syndrome of inappropriate antidiuretic hormone secretion (SIADH) (5, 30).

Pituitary apoplexy is an acute syndrome due to a sudden vascular damage of the pituitary gland, with hemorrhagic infarction and ischemia, mainly in the context of a preexisting pituitary macroadenoma. Frara et al. have well described patients with pituitary diseases in which COVID-19 caused pituitary apoplexy, as a plausible precipitating risk factor (30). Moreover, Gaudino et al. recently reported hypothalamic–pituitary failure also in a child with SARS-CoV-2 infection and suprasellar tumor (31). **Table 1** describes the clinical features and outcomes of cases of pituitary apoplexy in patients with SARS-CoV-2 infection.

On the other end, SIADH in COVID-19 seems due to several mechanisms related to systemic inflammation and pulmonary infection (17). In particular, a marked elevation of inflammatory cytokines can result in SIADH *via* two mechanisms. First, inflammatory cytokines (such as IL-6) can directly stimulate the no osmotic release of antidiuretic hormone (ADH) (32). Second, these cytokines can injure the lung tissue and alveolar cells, inducing SIADH *via* the hypoxic pulmonary vasoconstriction pathway (33). In patients with COVID-19, SIADH generally occurs with signs of moderate-severe hyponatremia (**Table 1**).

Few cases of central diabetes insipidus, central hypocortisolism, and rare findings of neurological symptoms with suspected radiological evidence of hypothalamic involvement have been described in patients with SARS-CoV-2 infection (**Table 1**).

### Case Report

A 36-year-old woman came for evaluation at the Department of Clinical and Experimental Sciences, Endocrine and Metabolic Unit, ASST Spedali Civili Brescia, University of Brescia (Italy) in September 2021, reporting secondary amenorrhea for 6 months (last menstruation in March 8, 2021). Prior to March 2021, she reported regular menstrual cycles, one physiological pregnancy (in 2012), and no comorbidities or long-term therapy, and blood exams showed eugonadism (last assessment in December 2019). In March 2021, the patient presented symptoms including slight fever, myalgias, fatigue, sore throat, and hyposmia and was diagnosed with SARS-CoV-2 infection by nasopharyngeal reverse transcriptase polymerase reaction (positive swab on March 15, 2021). These symptoms were spontaneously resolved in 12 days without treatment or hospitalization (negative swab on April 15, 2021). She was vaccinated against SARS-CoV-2 in September 2021.

In July 2021, the patient underwent a gynecological evaluation: physical examination and pelvic ultrasound were normal, she presented no clinical and biochemical signs of hyperandrogenism (normal values of adrenal androgens), blood exams suggested hypogonadotropic hypogonadism (**Table 2**), and the medroxyprogesterone acetate (MAP) test was negative; pregnancy, polycystic ovary syndrome (PCOS), and gynecological causes of amenorrhea were excluded.

**Table 2:** Blood exams.

Blood parameter	Data of dosage			n.v.
	July 2021	September 2021	November 2021	
Estradiol (ng/L)	<25	<25	<25	25–251
FSH (UI/L)	3.85	6.1	5.1	3–8
LH (UI/L)	0.29	<1	<1	1.8–11.78
PRL (mcg/L)	15.87	9	8	5–23
ACTH (pg/mL)	–	29	25	7–63
Cortisol (mcg/dL)	–	15.9	14.5	4.8–19.5
HGH (ng/mL)	–	0.84	–	–
IGF-1 (ng/mL)	–	133	–	43–286
TSH (mIU/L)	1.71	1.75	1.98	0.27–4.2
fT4 (ng/L)	–	8.92	9.1	9.3–17
Testosterone (µg/L)	0.16	0.16	–	0.08–0.48
Hemoglobin (g/dL)	13.6	–	–	12–16
Creatinine (mg/dL)	0.84	–	–	0.51–0.95
Glycemia (mg/dL)	62	–	–	60–100

n.v., normal values; FSH, follicle-stimulating hormone; LH, luteinizing hormone; PRL, prolactin; ACTH, adrenocorticotropic hormone; HGH, human growth hormone; IGF-1, insulin-like growth factor 1; TSH, thyrotropin-stimulating hormone; fT4, free thyroxine.

In August 2021, she underwent a dynamic enhanced MRI of the brain and pituitary region (given the finding of hypogonadotropic hypogonadism), showing a normal appearance of the sella turcica and regular dimensions of the adenohypophysis with uncertain millimetric (3 mm) pituitary microadenoma, and a normal appearance of the neurohypophysis, pituitary peduncle, median line structures, cavernous sinuses, optic chiasm, and supra- and subtentorial brain parenchyma. The FLAIR and DWI diffusive sequences of brain imaging did not report hypothalamic or other anormal findings.

At our endocrinological evaluation (September 2021), her temperature was 35.8°C, her pulse measured 72 beats per minute, and her blood pressure was 114/72 mmHg, weight 51 kg, height 1.58 m, and body mass index (BMI) 20.5 kg/m². She had no significant medical history, had no long-term therapy (no contraceptive pill or other drugs), and had never smoked. Besides amenorrhea, she presented no symptoms. Physical examination was normal. She reported no excessive physical training or significant fluctuations in body weight in recent years. The patient’s psychological state was carefully evaluated with particular care for stress related to the infection. The patient excluded any psychosocial state of stress.

On the suspicion of central amenorrhea, we carried out several blood tests (performed in the laboratory at our medical center): we excluded systemic disease (normal levels of inflammatory, coagulation, and hepatic markers), hemochromatosis (normal values of iron, ferritin, and transferrin saturation), celiac disease (negative anti-gliadin and anti-transglutaminase antibodies and no symptoms), overt primary thyroid disease, pituitary hypersecretion, adrenal axis deficit, and hyperprolactinemia, while we found low fT4 (free thyroxine) and confirmed hypogonadotropic hypogonadism, characterized in particular by low LH (luteinizing hormone) and estradiol with normal FSH (follicle-stimulating hormone) levels (**Table 2**).

Then, on the suspicion of central amenorrhea and possible central hypothyroidism, we performed a TRH test (intravenous infusion of 200 mcg thyrotropin-stimulating hormone) and a GnRH (gonadotropin-releasing hormone) analogue test (with subcutaneous triptorelin 0.1 mcg), on September 30, 2021, and October 7, 2021, respectively. These tests indicated a delayed pituitary response to TRH and a gonadotropin increase to the GnRH analogue, suggesting a hypothalamic deficiency (**Tables 3** and **4**). In particular, LH and FSH were suppressed and normal, respectively, at baseline and both normally increased acutely and after 24 h from triptorelin injection. Seven days after the TRH test, she presented euthyroidism with normal values of TSH (thyrotropin-stimulating hormone), fT4, and fT3 (free triiodothyronine) (**Table 3**). Ten days after the GnRH analogue test, the patient experienced menstrual spotting for a few days only. On November 3, 2021 (**Table 2**), hypogonadotropic hypogonadism persisted (still characterized by low LH and estradiol and normal FSH values), without resumption of the menstrual cycle. We plan to reevaluate the patient to decide the diagnostic–therapeutic follow-up, based on her interest in bearing children.

**Table 3:** TRH test.

Parameter	Time (minutes) after stimulation					
	0′ (at infusion)	+20′	+40′	+60′	+90′	+ 7 days
TSH (mIU/L)	2.14	13.90	14.20	16.70	16.30	2.27
fT4 (ng/L)						9.71
fT3 (ng/L)						2.3

TRH, thyrotropin-stimulating hormone; TSH, thyrotropin-stimulating hormone; n.v., normal values; fT4, free thyroxine; fT3, free triiodothyronine (n.v. 2.0–4.4 ng/l).

**Table 4:** GnRH analogous test.

Parameter	Time (minutes) after stimulation						
	0′ (at infusion)	+30′	+60′	+90′	+120′	+240′	+24 h
LH (UI/L)	<1	10.3	10.3	11.4	11.3	13.4	6.1
FSH (UI/L)	4.8	15.6	18.2	22.6	24.7	34.1	21.4
Estradiol (ng/L)	<25	–	–	–	–	–	55

GnRH, gonadotropin-releasing hormone; FSH, follicle-stimulating hormone; LH, luteinizing hormone.

## Discussion

We described a case of hypothalamic amenorrhea following COVID-19.

Recently, Phelan and colleagues have described that the COVID-19 pandemic could affect female reproductive health, causing dysmenorrhea, irregular menstrual cycle, and premenstrual symptoms through psychological distress (34). Nevertheless, to the best of our knowledge, no study has described female hypogonadotropic hypogonadism with secondary amenorrhea following SARS-CoV-2 infection.

Secondary amenorrhea is defined as the cessation of previously regular menses for 3 months or previously irregular menses for 6 months (35). In our patient, a diagnostic workup excluded all the known causes of secondary amenorrhea, in agreement to the current indications (35, 36). She had no psychological distress or physical factors for causing functional hypothalamic amenorrhea.

To understand whether the disorder was due to a hypothalamic deficiency (GnRH deficiency) or pituitary disease (doubtful microadenoma of 3 mm), we performed a TRH test, given the finding of low fT4, and an GnRH analogue test, even if these tests are not suggested in the diagnostic workup for amenorrhea (35).

The delay of pituitary response to TRH was observed in our patient (37), but the gonadotropin increase in response to GnRH analogue tests was suggestive for hypothalamic impairment (38, 39). Therefore, since our patient did not present any organic cause of hypothalamic–pituitary disorder, we hypothesized that her hypothalamic deficiency may have been an apparent consequence of SARS-CoV-2 infection.

This assumption, besides on the temporal consecutio, is strengthened by the fact that SARS-CoV-2 infection can impair the hypothalamic circuits, altering the endocrine axes, given that ACE2 receptors—which are responsible for the cells of this virus entering the cells—have also been observed in the hypothalamus (4, 5) and with a low concentration also in pituitary tumors (40). Indeed, ACE2 mRNA expression in the hypothalamus has been reported in *in vitro* and animal studies and confirmed in an autoptic human study (30). An excessive inflammation-mediated process at the hypothalamic–pituitary level (resulting in impairment or lesions in areas of the brain including the hypothalamic circuits) (28, 29) and a direct hypothalamic damage (due to SARS-CoV-2 neuroinvasion through the nasopharyngeal epithelium *via* the olfactory nerve or blood/brain barrier) (4, 27) are the two possible mechanisms for the hypothalamic dysfunction due to COVID-19 (2, 41). Even if our hypothesis seems strengthened by the above evidence, we must consider as major limitation the impossibility to directly demonstrate the presence of virus in the hypothalamic tissue *in vivo*. Otherwise, it is also true that this limitation is frequently present in many reports regarding the interaction between SARS-CoV-2 infection and glands (11, 26, 42).

This dysfunction could unfortunately be of considerable significance, given the extent of the COVID-19 pandemic. Firstly, this hypothalamic alteration could be underdiagnosed, passing unnoticed in postmenopausal women due to the absence of secondary amenorrhea as a warning sign. In addition, and of note, we cannot exclude a possible impact of this condition on female fertility. In our patient, despite recovery from infection and a partial resumption of menstrual spotting after GnRH analogue stimulation, the hypothalamic amenorrhea does not yet appear reversible, to the extent that we must consider possible reproductive consequences.

## Conclusion

We reported an unusual case of a young woman who developed hypothalamic amenorrhea following COVID-19. More cases are necessary to support the finding of hypothalamic amenorrhea as a complication of SARS-CoV-2 infection, also because it is difficult to directly demonstrate the presence of the virus in the hypothalamic tissue *in vivo*. Therefore, we suggest clinicians focusing greater attention and follow-up on possible existing or delayed endocrine disorders following COVID-19, in particular hypothalamic–pituitary dysfunction and female infertility. It would be desirable to consider and identify these possible complications and eventually direct the patient toward an adequate diagnostic–therapeutic management for female reproductive health.

## Data Availability Statement

The raw data supporting the conclusions of this article have been made available by the authors, without undue reservation.

## Ethics Statement

Written informed consent was obtained from the patient for the publication of any potentially identifiable images or data included in this article.

## Author Contributions

PF followed the patient, performed the literature review, wrote the first draft of the paper, and analyzed and critically discussed the results of the case report. VM followed the patient and wrote the first draft of the paper. AD wrote the first draft of the paper and analyzed and critically discussed the results of the case report. IP analyzed and critically discussed the results of the case report. MR analyzed and critically discussed the results of the case report. AF analyzed and critically discussed the results of the case report and the final version of the paper. CC followed the patient, coordinated the study, and analyzed and critically discussed the results of the case report and the final version of the paper. All authors contributed to the article and approved the submitted version.

## Conflict of Interest

The authors declare that the research was conducted in the absence of any commercial or financial relationships that could be construed as a potential conflict of interest.

## Publisher’s Note

All claims expressed in this article are solely those of the authors and do not necessarily represent those of their affiliated organizations, or those of the publisher, the editors and the reviewers. Any product that may be evaluated in this article, or claim that may be made by its manufacturer, is not guaranteed or endorsed by the publisher.
